# Impact of plant pathogens on potato rhizosphere enzymes and microbial dynamics

**DOI:** 10.3389/fpls.2025.1564704

**Published:** 2025-04-28

**Authors:** Gaihuan Xu, Linmei Deng, Jian Dao, Wenping Wang, Chunjiang Liu, Yanli Yang, Jing Zhao, Xia Liu

**Affiliations:** ^1^ Key Laboratory for Agro-biodiversity and Pest Control of Ministry of Education, College of Plant Protection, Yunnan Agricultural University, Kunming, China; ^2^ Institute of Agricultural Environment and Resources, Yunnan Academy of Agricultural Sciences, Kunming, China

**Keywords:** soil-borne diseases, stress, soil enzyme activity, soil microorganisms, potato

## Abstract

Soilborne pathogens significantly impact potato productivity by altering rhizosphere enzymatic activities and microbial communities. Pathogen-induced changes in enzyme activities are correlated with shifts in microbial community composition, but causal relationships remain unclear. This study investigates the effects of five key pathogens—*Phytophthora infestans*, *Streptomyces scabies*, *Spongospora subterranea*, *Ralstonia solanacearum* and *Globodera rostochiensis*—on soil enzyme activities and microbial community structure in potato rhizosphere soils under continuous cropping. This experiment involved pathogen inoculation and soil sampling in potato rhizosphere soils, with treatments replicated three times. Potatoes were planted on March 22, 2023, and harvested on August 25, 2023. Enzymatic activities were measured at different growth stages, and microbial communities were analyzed using high-throughput sequencing. Pathogen-induced variations in enzymatic activities were observed, potentially promoting disease proliferation. For instance, under *S. scabies* stress, urease (URE) activity increased significantly at the full flowering and post-flowering stages, while catalase (CAT) activity decreased significantly during the seedling and full flowering stages. Under *S. subterranea* stress, activities of urease, sucrase (SUC), and alkaline phosphatase (ALP) decreased. *M. nematode* stress led to a decline in URE and sucrase activities. *P. infestans* infection led to a decrease in URE activity at the sowing stage. Furthermore, microbial community composition was significantly correlated with disease incidence, with specific taxa such as *Planctomycetes* and *Basidiomycota* showing negative correlations with *S. subterranea* incidence, while *Candidatus Dormibacteraeota* and *Ascomycota* were positively associated with *P. infestans*. These results suggest that pathogen-induced changes in enzymatic activities play a critical role in disease dynamics and microbial interactions. The findings highlight the importance of understanding the effects of soilborne pathogens on soil enzyme activities and microbial communities, providing insights into disease management strategies in potato farming.

## Introduction

Potato (*Solanum tuberosum* L.), a member of the Solanaceae family, is an annual crop renowned for its resilience to cold, drought tolerance, adaptability to nutrient-poor soils, and broad ecological versatility (Xu et al., 2019). As the third most significant staple crop worldwide, the potato plays a crucial role in enhancing food security (FAO data, https://www.fao.org/statistics/en/). Although China is the largest producer, its average potato yield over the past decade has been only 16.4 t/ha, significantly below the global average. This yield gap is attributed not only to limitations in natural resources, but also to the growing impact of pests and diseases, which remain a major bottleneck for improving potato productivity in the country (Xu et al., 2019). The prevalence of traditional potato diseases, such as late blight, early blight, and bacterial wilt, has been increasing steadily each year. Potato late blight (*Phytophthora infestans*) poses a significant threat to potato production in China, affecting approximately 2.05 million hectares annually across various ecological cultivation zones. In years of severe epidemics, the disease can impact more than 46% of the sown area, leading to yield losses of 10%–30%, and in extreme cases, exceeding 50% or even resulting in total crop failure (Huang and Liu, 2016a). Early blight (*Alternaria solani*), the second most prevalent potato disease in China after late blight, has exhibited an increasing trend in severity. Currently, the disease affects approximately 900,000 hectares nationwide (Huang and Liu, 2016b). Bacterial wilt, caused by *Ralstonia solanacearum*, is a globally significant plant disease (Xu and Feng, 2013). In China, potato bacterial wilt caused by *R. solanacearum* Race 3/Biovar II (r3bv2) is widely distributed across major potato-producing regions, except for the northeastern zone. The average disease incidence ranges from 5% to 20%, with extreme outbreaks reaching over 90% (Jiang et al., 2017). Meanwhile, previously sporadic secondary diseases, including black scurf, *Fusarium* wilt, common scab, powdery scab, and blackleg, have become major threats, leading to localized outbreaks with devastating impacts (Xu et al, 2019). Soilborne diseases are a serious concern in China’s potato production, affecting approximately 437,000 hectares annually (Xu et al., 2019). The average disease incidence rate is between 5%-30%, and in extreme cases, it can be as high as over 90% (Andrade et al., 2019; Jia et al., 2023; Zhang et al., 2018, 2012; Sun et al., 2024; Zhang, 2014). While *P. Infestans* is primarily known as a foliar pathogen, it can also persist in soil under certain conditions, particularly through infected plant debris. We focused on its secondary effects on soil microbial communities and enzyme activities following foliar infection (Pang et al., 2021). The plant root system, along with surrounding soil and microbial communities, form a complex rhizosphere ecosystem, characterized by intricate and dynamic interactions among its components (Singh et al., 2011). The interactions within this ecosystem between the roots, soil, and microorganisms are crucial for maintaining soil health and disease resistance (Chen and Liu, 2024). Many researchers have explored the relationship between rhizosphere microbial diversity and soil-borne disease occurrence from an ecological perspective. Comparisons of soil bacterial communities from two natural banana *Fusarium* wilt-suppressive orchards showed that these disease-suppressive soils harbored distinctive bacterial communities, compared to their disease-conducive counterparts. Through *in vitro* culture and confrontation assays, reveals that banana *Fusarium* wilt suppressive soils harbor unique communities with higher richness and diversity and identified specific populations that may be considered as indicators of the ability of a soil to suppress disease (Shen et al. 2015).

Plant roots influence rhizosphere soil properties and reshape microbial community structures through nutrient exudation. Plant roots influence the physical and chemical properties of rhizosphere soil and reshape microbial community structures through nutrient exudation (Berg and Smalla, 2009). Soil enzymes, as the most biologically active organic components in soil, serve as catalysts for various biochemical processes, including organic matter decomposition, nutrient cycling, and energy transfer. These enzymes are widely regarded as key indicators of soil health and fertility. The diversity of soil enzymes includes hydrolases, oxidoreductases, transferases, lyases, isomerases and ligases, with urease, phosphatase, sucrase, and catalase playing pivotal roles in nitrogen, phosphorus, and carbon nutrient cycling, as well as in plant protection (Li et al., 2019; Carvalho et al., 2019). Research further indicates that most soil bacteria and fungi can produce enzymes such as pectinase, amylase, and cellulase (Sun et al., 1997). Additionally, *Fusarium oxysporum*, a notable fungal pathogen, is known to secrete lipase into the soil environment (Liu et al., 2011). Significant correlations have also been observed between microbial activity and community structure in rhizosphere soil and the activity of key enzymes, including urease (Chakrabarti et al., 2004), alkaline phosphatase, cellulase (Liu et al., 2021), sucrase, and catalase (Cui et al., 2020).

Soil enzyme activity is a sensitive indicator of soil health and fertility, as it facilitates the transformation of organic matter and nutrient cycling (You et al., 2014). Soil enzymes facilitate the transformation of organic matter and nutrient cycling; however, disruptions in their activity can impede soil metabolic processes (Yan et al., 2019). Such disruptions may alter the micro-ecological environment of the plant rhizosphere and root system, potentially influencing the accumulation of soilborne pathogens. For example, studies by You et al. (2014) reported a significant reduction in sucrase, dehydrogenase, urease, and phosphatase activities in the rhizosphere soil of *Panax notoginseng* infected with root rot, with these enzymatic changes being correlated with disease severity (Lorenz et al., 2020). In highland barley infected with root rot, sucrase, alkaline phosphatase, and urease activities in the soil were significantly reduced, whereas cellulase activity increased, and catalase activity showed no significant change (Li et al., 2017). As shown, soil enzyme activity and microbial dynamics are closely linked to various crop diseases, including those that affect potatoes. Given the growing challenges posed by rapidly evolving pathogens, increasing resistance, and the growing demand for sustainable agricultural practices, it is critical to explore new strategies not only for improving yields but also for achieving broader agricultural goals, such as controlling soilborne diseases. Effective management of these diseases is crucial for the implementation of agricultural “double reduction” initiatives and ensuring food security, which aims to reduce fertilizer and pesticide use in agriculture (Cerda et al., 2017). Investigating the relationship between soil enzyme activity and plant diseases offers valuable insights into how micro-ecological regulation can be used to manage soil-borne diseases in potato cultivation.

Therefore, investigating the relationship between soil enzyme activity and plant disease is of significant importance. High-throughput sequencing and other advanced technologies enable the analysis of factors contributing to soil-borne disease occurrence at the microbial community level, facilitating the identification of efficient and environmentally friendly biological control strategies (Glasa et al., 2019). This study aims to analyze changes in enzyme activity under pathogen stress and their relationships with soilborne disease occurrence. Understanding these relationships will provide a theoretical basis for employing micro-ecological approaches to control soilborne diseases, which could significantly enhance potato cultivation practices and contribute to the sustainable development of the industry. The study addresses critical gaps in understanding how plant pathogens influence rhizosphere enzyme activities and microbial dynamics in potato crops, which is essential for developing sustainable disease management strategies.

## Materials and methods

### Five distinct methods for pathogen inoculation testing

The potato cultivar ‘Hezuo 88,’ a widely cultivated variety in Yunnan Province, was used in this study. All pathogens were obtained from the Potato Disease Laboratory at Yunnan Agricultural University, including *Streptomyces scabies* causing common scab, *Spongospora subterranea* f. sp. *subterranea* causing powdery scab, *Ralstonia solanacearum* causing bacterial wilt, *Phytophthora infestans* causing late blight, and *Globodera rostochiensis*, a cyst nematode of potato.

To prepare the pathogen inocula, the following methods were used depending on the organism. The selected pathogens were introduced into the planting furrows as a soil drench at a concentration of approximately 150 grams per plant during planting. The inoculum was prepared by culturing each pathogen in a suitable medium, and the final concentration was adjusted to 1 × 10^-6^ CFU/mL. The soil drench method was chosen to ensure uniform distribution of the pathogens in the rhizosphere. Control plants were treated with sterile water under the same conditions.

1) *Streptomyces* scabies: cultivated on modified Gause’s medium No.1 (Hu et al., 2020) and incubated at 28°C for 7 to 9 days. The colonies on plates were scraped and diluted to a concentration of 1 × 10^8^ CFU·mL^-1^ in sterile water. 2) *Spongospora subterranea*: infected potato tuber skin was peeled and homogenized in sterile water and adjusted to 1 × 10^8^ CFU·mL^-1^ based on spore counts under a microscope, using a sterile mortar and pestle. 3) *Globodera rostochiensis*: 100 g of soil containing cyst nematodes was mixed with 1 L of water, allowed to settle for 30 seconds, and filtered through 80- and 200-mesh sieves. The cysts were rinsed onto filter paper, and 20 cysts per 100 g soil were prepared for inoculation. 4) *Phytophthora infestans*: *P. infestans* was cultivated on rye-tomato medium (Qian et al., 2021) at 19°C for 12 to 15 days. Following this period, the mycelium was gently scraped, suspended in sterile water, and adjusted to a concentration of 5 × 10^4^ sporangia per mL. The prepared suspension was then stored at 4°C to promote zoospore release. 5) *Ralstonia* suspension: *R. solanacearum* was cultured in beef extract-peptone medium (Shen et al., 2018) at 28°C with shaking at 120 rpm for 48 hours. The suspension was adjusted to an OD of 0.1 (1 × 10^8^ CFU·mL^-1^) using a UV spectrophotometer.

The community trial adopted a randomized block design, with 6 treatments set up, each process handles 3 replicates, for a total of 18 plots. The residential area is 32 square meters (length 8 m, width 4 m), with a row spacing of 70 cm and a plant spacing of 32 cm, planting 150 potato plants.

1: untreated control (CK); 2: *Streptomyces* scabies (CJ); 3: *Spongospora subterranea* (FJ); 4: *Globodera rostochiensis* (JXC); 5: *Phytophthora infestans* (WY); and 6: *Ralstonia solanacearum* (QK). Each treatment was replicated three times, resulting in a total of 18 plots. Pathogens were introduced into the planting furrows as a soil drench at approximately 150 grams per plant during planting on March 22, 2023, and harvested on August 25, 2023. Potatoes were cultivated following standard agricultural practices, apply potato-specific fertilizer (N:P:K=10:8:9) during sowing. Soil samples were collected at the full flowering stage and analyzed using Illumina MiSeq sequencing.

### Enzyme activity of soil samples collected from potato rhizosphere

Soil samples were collected at six key growth stages: planting stage(PS), seedling stage(SS), initial flowering stage(IS), full flowering stage(FS), end flowering stage(ES), and harvest stage(HS). The activities of urease, sucrase, alkaline phosphatase, and catalase were assessed using standard protocols, including the phenol-hypochlorite method, the 3,5-dinitrosalicylic acid method, the disodium phenyl phosphate method (Lorenz et al., 2020), and UV spectrophotometry that catalase activity was measured based on the rate of H_2_O_2_ decomposition at 240 nm (Yang et al., 2011), respectively. Rhizosphere soil sampling includes the distance from the root (0-5 mm), depth (0-20 cm), and mixing method (mixing 5 samples from each plot). The samples are stored in a -80°C freezer for future use. Each treatment was replicated three times, planting on March 22, 2023, and harvested on August 25, 2023.

### Soil microbial high-throughput sequencing

Soil DNA was extracted using the E.Z.N.A.^®^ Soil DNA Kit (Omega Bio-tek, Norcross, GA, USA). The bacterial 16S rRNA V1–V9 regions were amplified with primers 27F (5’-AGRGTTYGATYMTGGCTCAG-3’) and 1492R (5’-RGYTACCTTGTTACGACTT-3’), while fungal ITS1–ITS4 regions were amplified using primers ITS1F (5’-CTTGGTCATTTAGAGGAAGTAA-3’) and ITS4R (5’-TCCTCCGCTTATTGATATGC-3’).

The PCR reaction mixture included 4 μL of 5× FastPfu Buffer, 2 μL of 2.5 mM dNTPs, 0.8 μL of each primer, 0.4 μL of FastPfu Polymerase, and 10 ng of template DNA, with sterile ddH_2_O added to a final volume of 20 μL. The PCR conditions were as follows: initial denaturation at 95°C for 2 minutes, followed by 27 cycles of denaturation at 95°C for 30 seconds, annealing at 55°C for 30 seconds, and extension at 72°C for 1 minute for the 16S rRNA amplification; for ITS region amplification, 39 cycles were applied. The final extension was performed at 72°C for 5 minutes, and the products were then held at 10°C. Amplicons were purified using the AxyPrep DNA Gel Extraction Kit (Axygen Biosciences, Union City, CA, USA) and sequenced on an Illumina MiSeq platform (Kyoungwon, et al., 2016) by Shanghai LingEn Biotechnology Co., Ltd (Shanghai, China).

### Data analysis

Data were processed and analyzed using several software tools: Excel for preliminary calculations, DPS for single-factor ANOVA, and GraphPad Prism 9.0 for visualization. Paired-end sequence data were processed using SMRT Link Analysis software (version 6.0) to generate circular consensus sequencing (CCS) reads. Raw reads were filtered to remove low-quality sequences, duplicates, and those not meeting specified length and quality thresholds, yielding high-quality sequences for each sample. Operational Taxonomic Units (OTUs) were clustered at a similarity threshold of 98.65% using the UPARSE algorithm. We employed one-way analysis of variance (ANOVA) to assess significant differences among treatments, followed by Tukey’s honest significant difference (HSD) test for multiple comparisons. For non-normally distributed data, we used the Kruskal-Wallis test, followed by Dunn’s *post hoc* test. These statistical analyses were performed using SPSS software, with a significance threshold set at p < 0.05.

The composition and diversity of the bacterial and fungal communities (both Alpha and Beta diversity) were analyzed at various taxonomic levels using tools such as Mothur (https://mothur.org/), NCBI Blast (https://blast.ncbi.nlm.nih.gov/Blast.cgi), and PICRUSt2 (https://github.com/picrust/picrust2/wiki/Key-Limitations) for functional predictions.

## Results

### Establishment of the standard curve

**Figure 1:**
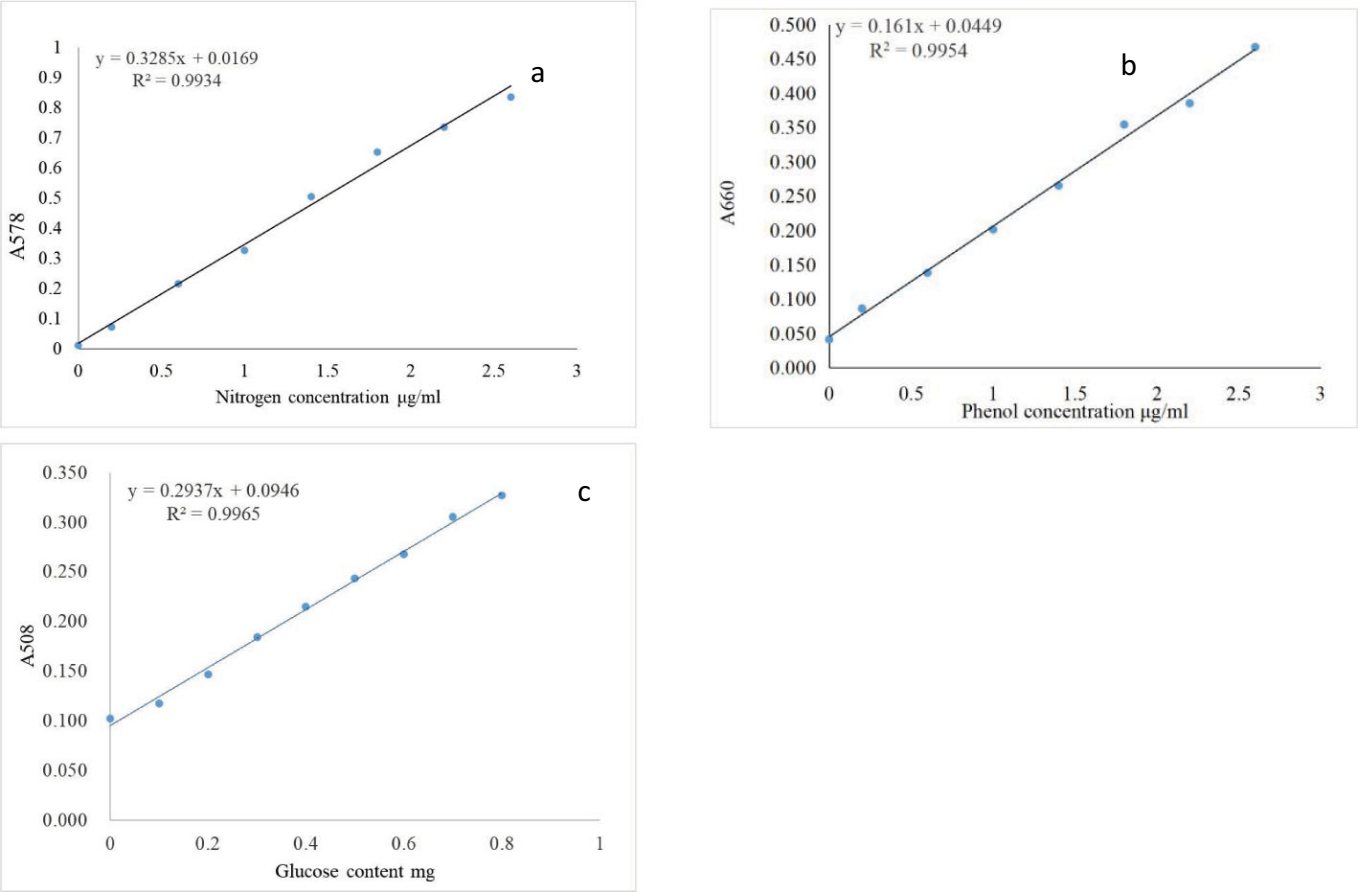
Standard curves of absorbance for nitrogen at 578 nm **(a)**, phenol at 660 nm **(b)** and glucose at 508 nm **(c)** to measure enzymatic activities.

The standard curve was constructed with nitrogen concentration, glucose content, and phenol concentration as the independent variables, with the corresponding absorbance values plotted on the dependent variable. The results showed that the correlation coefficient for each equation exceeded 0.99, indicating a strong linear relationship (**Figure 1**).

### Soil urease activity

**Figure 2:**
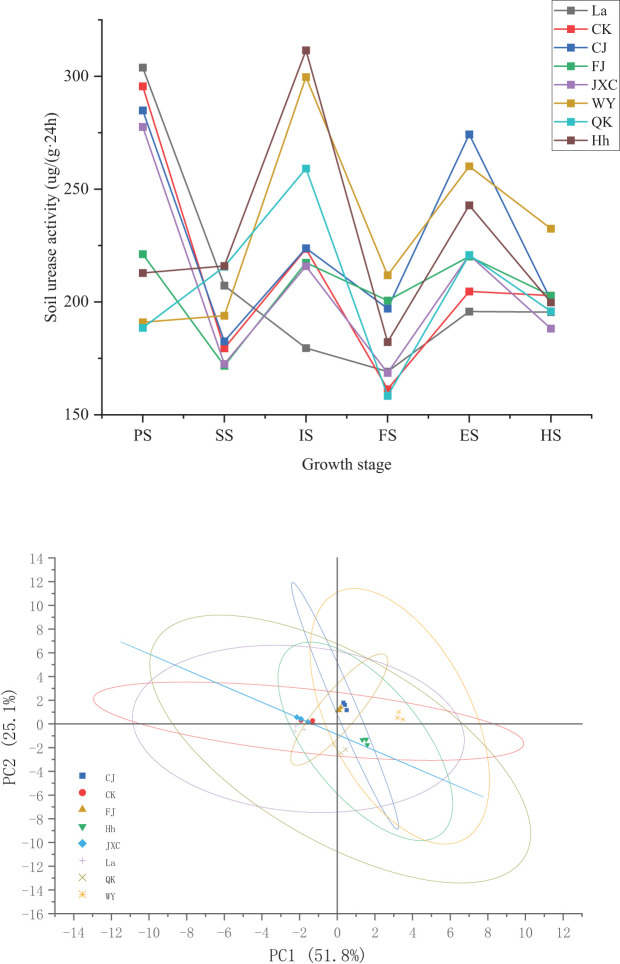
Soil urease activity in rhizosphere soil during the potato growth stages soil urease activity under different treatments. Treatments include the control (CK), *Streptomyces scabies*, (CJ), *Spongospora subterranea* (FJ), *Globodera rostochiensis* (JXC), *Phytophthora infestans* (WY), and *Ralstonia solanacearum* (QK). Growth stage: planting stage (PS), seedling stage (SS), initial flowering stage (IS), full flowering stage (FS), end flowering stage(ES), and harvest stage (HS).

Urease activity exhibited punctuations at different stages of potato growth across various treatments, including the control (CK), *Streptomyces scabies* (CJ), *Spongospora subterranea* (FJ), and *Globodera rostochiensis* (JXC). Generally, urease activity increased from sowing to harvest, with distinct patterns observed in the pathogen treatments. In the WY (late blight) and QK (bacterial wilt) treatments, urease activity increased steadily from sowing to the initial flowering stage, followed by a further rise during the end flowering stage. Compared to CK, the CJ treatment (common scab) significantly enhanced urease activity by 22.1% and 34.0% at the full and end flowering stages, respectively. In the FJ treatment (powdery scab), urease activity initially decreased at sowing but subsequently increased to 200.60 and 220.19 μg·(g·24h)^-1^ at full bloom and flowering decline, respectively. Under the JXC treatment, urease activity declined from 295.58 to 277.59 μg·(g·24h)^-1^ and from 202.83 to 188.25 μg·(g·24h)^-1^ at sowing and harvest stages, respectively, while an increase from 161.36 to 168.59 μg·(g·24h)^-1^ was observed during full bloom. In the WY treatment, urease activity decreased by 104.57 μg·(g·24h)^-1^ at sowing relative to the control, followed by subsequent increases of 14.44, 76.13, 50.51, 55.45, and 29.69 μg·(g·24h)^-1^ from seedling through harvest. In the QK treatment, urease activity decreased by 107.04 μg·(g·24h)^-1^ at sowing but then increased by 36.20, 35.57, and 16.14 μg·(g·24h)^-1^ during the seedling, early flowering, and flowering decline stages, respectively, with significant differences observed (**Figure 2**). Urease was enhanced by *Streptomyces scabies* (CJ), *Phytophthora infestans* (WY), and *Ralstonia solanacearum* (QK) stresses, while reduced by *Spongospora subterranea* (FJ) and *Globodera rostochiensis* (JXC) tresses.

### Soil URE activity

**Figure 3:**
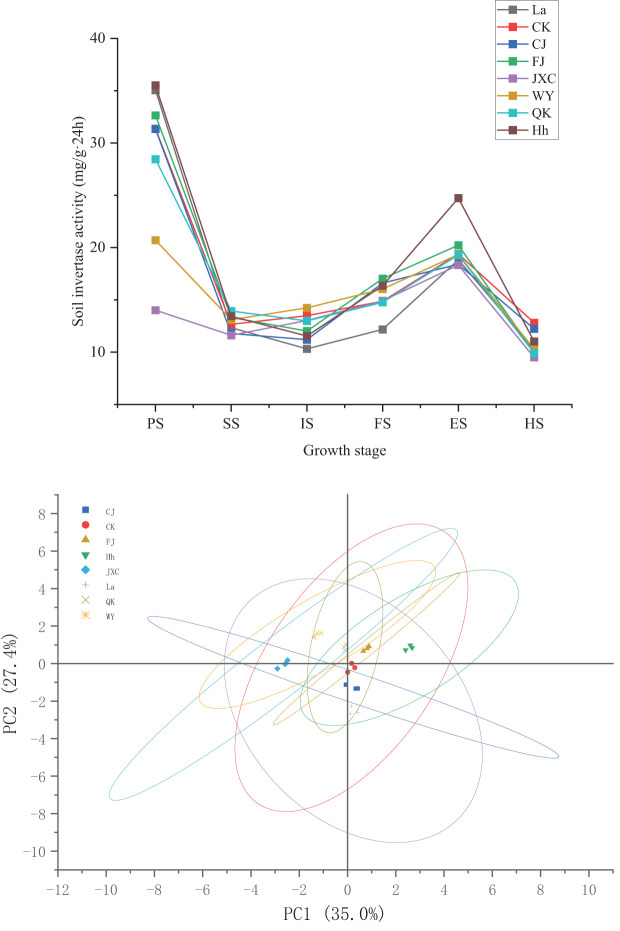
Soil sucrase activities in rhizosphere soil under different treatments during the potato growth period. Treatments include the control (CK), *Streptomyces scabies*, (CJ), *Spongospora subterranea* (FJ), *Globodera rostochiensis* (JXC), *Phytophthora infestans* (WY), and *Ralstonia solanacearum* (QK). Growth stage: planting stage (PS), seedling stage (SS), initial flowering stage (IS), full flowering stage (FS), end flowering stage (ES), and harvest stage (HS).

Throughout the growth phases of potato plants, soil urease (SUC) activity in the rhizosphere followed a consistent pattern across both the control (CK) and treatments involving various soilborne pathogens. SUC activity decreased during the seedling stage, increased from early to late flowering, and declined at harvest. In the CJ treatment (common scab), SUC activity was reduced by 0.89, 2.29, 1.02, and 0.57 mg·(g·24h)^-1^ during the seedling, early flowering, flowering decline, and harvest stages, respectively, compared to the control (CK). However, a significant increase of 1.74 mg·(g·24h)^-1^ was observed during the full bloom stage. The FJ treatment (powdery scab) showed marked increases in SUC activity of 1.28, 0.65, 2.16, and 0.83 mg·(g·24h)^-1^ during the sowing, seedling, full bloom, and flowering decline stages, representing changes of 4.1%, 5.1%, 14.5%, and 4.3%, respectively. However, reductions of 1.47 mg·(g·24h)^-1^ and 2.78 mg·(g·24h)^-1^ were observed during the early flowering and harvest stages, with respective decreases of 10.9% and 21.7%. Under the JXC treatment (cyst nematodes), SUC activity remained relatively stable during the full bloom stage but significantly decreased at sowing, seedling, and early flowering stages from 31.36, 12.67, and 13.49 mg·(g·24h)^-1^ to 14.01, 11.62, and 13.03 mg·(g·24h)^-1^, respectively. Further reductions in nutrient uptake rates, from 19.39 and 12.81 to 18.33 and 9.51 mg·(g·24h)^-^¹ during the flowering decline and harvest stages, respectively, represented decreases of 55.3%, 8.3%, 3.4%, 5.4%, and 25.7% compared to the control (CK). In the WY treatment (late blight), SUC activity decreased from 31.36 and 12.81 to 20.71 and 10.30 mg·(g·24h)^-1^ during sowing and harvest, while it increased from 13.49 and 14.87 to 14.24 and 16.03 mg·(g·24h)^-1^ during early flowering and full bloom, with significant differences between these periods. For the QK treatment (bacterial wilt), soil SUC activity declined by 2.91, 0.50, and 2.90 mg·(g·24h)^-1^ at the sowing, early flowering, and harvest stages, respectively. In contrast, an increase of 1.26 mg·(g·24h)^-1^ was noted during the seedling stage, all changes being statistically significant (**Figure 3**).

The findings reveal that stress from *Streptomyces scabies* (CJ) significantly influences soil SUC activity during the seedling stage, although overall fluctuations were minimal. In contrast, *Spongospora subterranea* (FJ) stress consistently reduced SUC activity, particularly during the seedling, full bloom, and harvest stages. *Globodera rostochiensis* (JXC) also notably impacted SUC activity, especially during the seedling and harvest stages. Exposure to *Phytophthora infestans* (WY) stress initially enhanced SUC activity during the early flowering stage but ultimately resulted in a decrease by harvest, with no significant overall variation. Lastly, *Ralstonia solanacearum* (QK) stress contributed to a reduction in SUC activity at harvest, though overall changes remained subtle.

### Soil ALP activity

**Figure 4:**
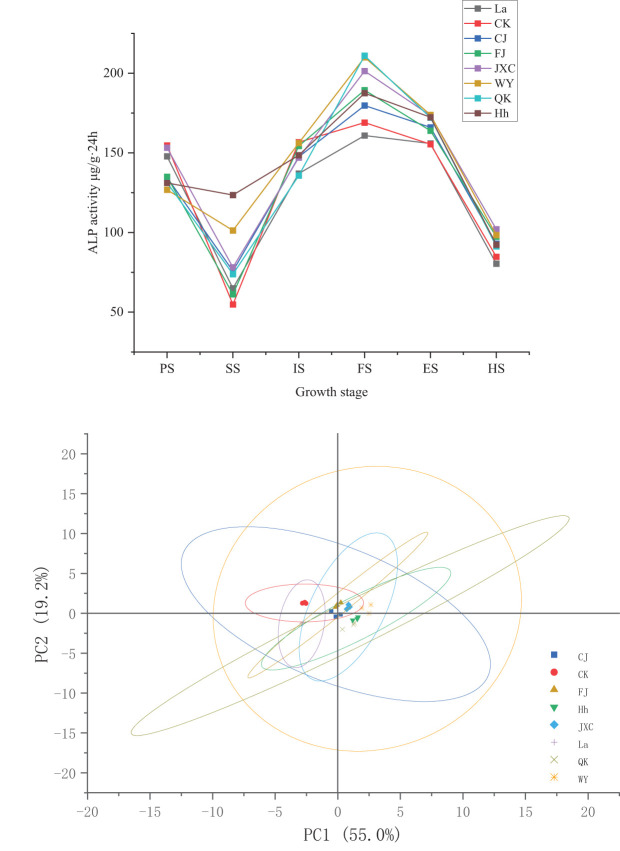
Variation in soil alkaline phosphatase activities in rhizosphere soil under different treatments during the potato growth period. Treatments include the control (CK), *Streptomyces scabies*, (CJ), *Spongospora subterranea* (FJ), *Globodera rostochiensis* (JXC), *Phytophthora infestans* (WY), and *Ralstonia solanacearum* (QK). Growth stage: planting stage (PS), seedling stage (SS), initial flowering stage (IS), full flowering stage (FS), end flowering stage (ES), and harvest stage (HS).

During the growth period of potato plants, alkaline phosphatase (ALP) activity in the rhizosphere soil exhibited a consistent pattern across both the control (CK) and various soilborne pathogen treatments. ALP activity initially decreased during the seedling stage, peaked at full bloom, and then declined steadily from the flowering decline stage onwards. Under the CJ treatment, compared to CK, ALP activity decreased by 20.13 and 9.18 μg·(g·24h)^-1^ during the sowing and early flowering stages, respectively, compared to the control (CK). However, significant increases of 21.02, 10.66, 10.58, and 8.19 μg·(g·24h)^-1^ were observed during the seedling, full bloom, flowering decline, and harvest stages. In the FJ treatment (powdery scab), ALP activity remained stable during the early flowering stage, with significant increases of 6.41, 20.38, 8.43, and 12.53 μg·(g·24h)^-1^ recorded during the seedling, full bloom, flowering decline, and harvest stages. These increases corresponded to percentage increases of 11.7%, 12.1%, 5.4%, and 14.8%, respectively. For the JXC treatment (cyst nematodes), ALP activity decreased notably by 9.69 μg·(g·24h)^-1^ during the early flowering stage. However, significant increases of 23.33, 32.36, 17.96, and 17.25 μg·(g·24h^)-1^ were observed during the seedling, full bloom, flowering decline, and harvest stages, representing percentage differences of 6.2%, 42.6%, 19.1%, and 20.3%, respectively. For the WY treatment (late blight), ALP activity decreased by 27.83 μg·(g·24h)^-1^ from 154.66 to 126.83 μg·(g·24h)^-1^ during the sowing stage. In contrast, increases of 46.48, 40.96, 18.38, and 13.57 μg·(g·24h)^-1^ were recorded during the seedling, full bloom, flowering decline, and harvest stages, corresponding to percentage changes of 84.8%, 24.2%, 11.8%, and 16.0%, respectively. In the QK treatment, ALP activity was reduced by 23.35 and 20.94 μg·(g·24h)^-1^ during the sowing and early flowering stages, respectively. Conversely, significant increases of 19.06, 41.96, and 16.67 μg·(g·24h)^-1^ were observed during the seedling, full bloom, and flowering decline stages, with notable percentage differences of 15.1%, 13.4%, 34.8%, 10.9%, and 10.7% (**Figure 4**).

These results demonstrate that the common scab pathogen (CJ) caused notable fluctuations in ALP activity in the soil during the seedling and full bloom stages, although the overall impact was moderate. In contrast, stress from the powdery scab pathogen (FJ) primarily led to reductions in ALP activity during the seedling and early flowering stages. Nematode (JXC) infestation significantly enhanced ALP activity, particularly during the seedling and full bloom stages. Similarly, exposure to stress from the late blight pathogen (WY) resulted in increased ALP activity, especially during the seedling and flowering decline stages. Conversely, bacterial wilt pathogen (QK) stress suppressed soil ALP activity, with the most pronounced reductions occurring during the full bloom and flowering decline stages.

### Soil CAT activity

**Figure 5:**
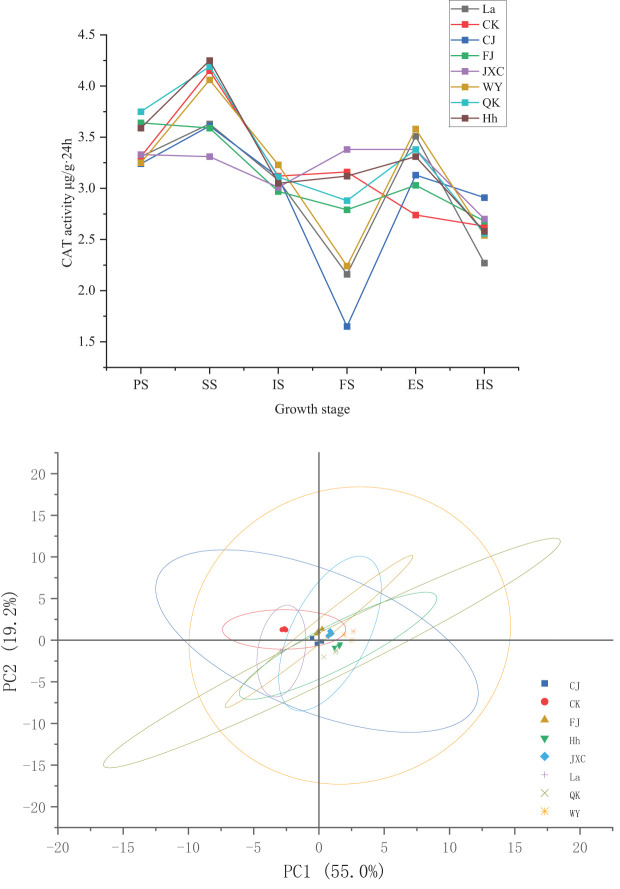
Dynamics of soil catalase activity in rhizosphere soil under different treatments during the potato growth period. Treatments include the control (CK), *Streptomyces scabies*, (CJ), *Spongospora subterranea* (FJ), *Globodera rostochiensis* (JXC), *Phytophthora infestans* (WY), and *Ralstonia solanacearum* (QK). Growth stage: planting stage (PS), seedling stage (SS), initial flowering stage (IS), full flowering stage (FS), end flowering stage (ES), and harvest stage (HS).

During the potato growth period, changes in catalase (CAT) activity in the rhizosphere soil across both the control (CK) and various soilborne pathogen treatments were minimal, with CAT activity peaking during the seedling stage. Compared to CK, CAT activity in the rhizosphere soil of potato plants significantly decreased by 0.54 and 1.51 mg·(g·20min)^-1^ during the seedling and full bloom stages, respectively, while increasing by 0.39 mg·(g·20min)^-1^ during the flowering decline stage. These changes corresponded to percentage differences of 14.2%, 13.0%, and 48.0%, respectively. Under the FJ treatment, CAT activity significantly decreased by 0.56 and 0.37 mg·(g·20min)^-1^ during the seedling and full bloom stages, while showing significant increases of 0.33 and 0.29 mg·(g·20min)^-1^ during the sowing and flowering decline stages. These variations corresponded to percentage differences of 13.5%, 11.9%, 8.9%, and 10.6%, respectively. For the JXC treatment, CAT activity declined from 4.15 to 3.31 mg·(g·20min)^-1^ during the seedling stage. However, increases were observed during the full bloom and flowering decline stages, rising from 3.16 and 2.74 mg·(g·20min)^-1^ to 3.38 mg·(g·20min)^-1^, representing significant increases of 7.0% and 23.5% compared to the control. In the WY treatment, CAT activity decreased by 0.92 mg·(g·20min)^-1^ during the full bloom stage but increased by 0.84 mg·(g·20min)^-1^ during the flowering decline stage, corresponding to percentage differences of 29.2% and 30.7%, respectively. Similarly, under the QK treatment, CAT activity decreased by 0.28 mg·(g·20min)^-1^ during the full bloom stage and increased by 0.64 mg·(g·20min)^-1^ during the flowering decline stage, reflecting percentage changes of 8.9% and 23.5%, respectively (**Figure 5**).

These results demonstrate that common scab pathogen (CJ) primarily reduced soil CAT activity during the seedling and full bloom stages. In contrast, the powdery scab pathogen (FJ) affected CAT activity during the seedling, full bloom, and flowering decline stages, although its overall impact on CAT activity was relatively modest. The presence of nematodes (JXC) increased CAT activity, particularly during the full bloom stage, though the effect remained minimal over the entire growth period. Exposure to the late blight pathogen (WY) altered CAT activity during the full bloom stage but had limited influence throughout the overall growth cycle. Finally, bacterial wilt pathogen (QK) significantly enhanced soil CAT activity, especially during the full bloom and flowering decline stages.

In summary, the presence of soilborne pathogens led to varying degrees of alteration in soil URE, SUC, ALP, and CAT activities. Specifically, the common scab pathogen (CJ) significantly increased URE activity while decreasing CAT activity in the rhizosphere soil during potato growth, with no notable changes observed in SUC and ALP activities. Powdery scab pathogen (FJ) stress significantly reduced URE, SUC, and ALP activities, while CAT activity remained relatively unaffected. Nematode (JXC) presence significantly decreased URE and SUC activities while increasing ALP activity, with minimal impact on CAT activity. Late blight pathogen (WY) stress markedly enhanced URE and ALP activities in the soil, with limited effects on SUC and CAT activities. Lastly, bacterial wilt pathogen (QK) stress significantly increased URE and CAT activities, while reducing ALP activity and having minimal impact on SUC activity.

### Phylum-level composition and structural characteristics of bacterial communities in potato rhizosphere soil

**Figure 6:**
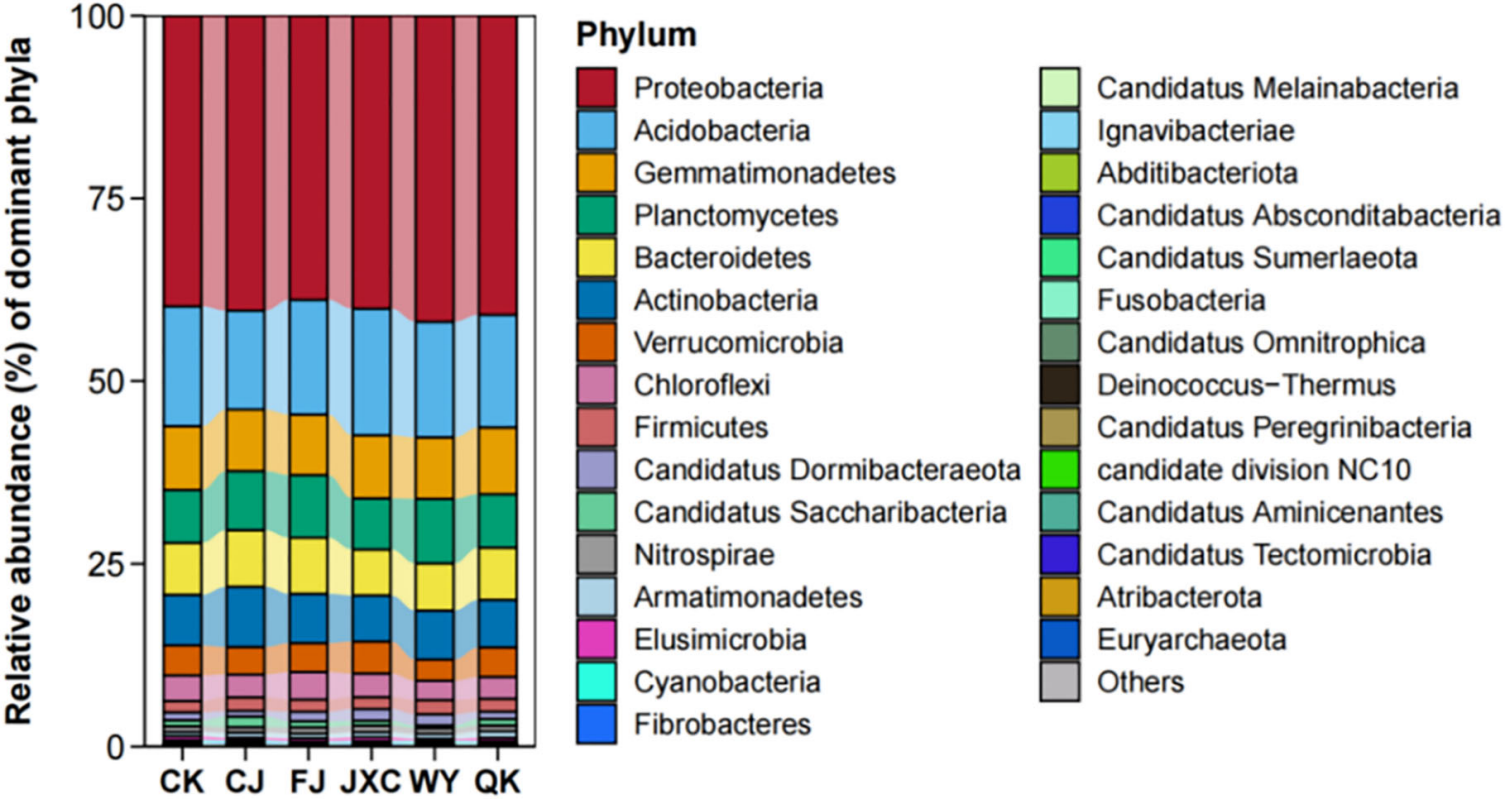
Relative abundance of bacterial communities at the phylum level in potato rhizosphere soil during the full flowering stage genus-level composition and structural dynamics of bacterial communities n potato rhizosphere soil. Treatments include the control (CK), *Streptomyces scabies*, (CJ), *Spongospora subterranea* (FJ), *Globodera rostochiensis* (JXC), *Phytophthora infestans* (WY), and *Ralstonia solanacearum* (QK).

The microbial profiles of rhizosphere microorganisms across different taxonomic levels are presented in the sequence of treatments: ‘CK,’ ‘CJ,’ ‘FJ,’ ‘JXC,’ ‘WY,’ and ‘QK.’ At the phylum level, the number of bacterial phyla identified was 29, 29, 32, 29, 34, and 32, respectively, indicating that soilborne pathogen treatments slightly increased bacterial diversity in the potato rhizosphere, although these differences were not statistically significant. The top 10 dominant bacterial phyla were consistent across all groups and included Proteobacteria, Acidobacteria, Gemmatimonadetes, Planctomycetes, Bacteroidetes, Actinobacteria, Verrucomicrobia, Chloroflexi, Firmicutes, and Candidatus Dormibacteraeota. Among these, Proteobacteria was the most abundant phylum, with relative abundances of 39.78%, 40.37%, 38.88%, 40.09%, 41.88%, and 40.94% in the CK, CJ, FJ, JXC, WY, and QK groups, respectively. Acidobacteria was the second most abundant phylum, with relative abundances ranging from 13.52% to 16.38%. The phylum Gemmatimonadetes was the third most abundant phylum in the CK (control), CJ, JXC, and QK treatments, with relative abundances of 8.74%, 8.42%, 8.63%, and 9.13%, respectively. Additionally, it ranked as the fourth most abundant phylum in the FJ and WY treatments, with relative abundances of 8.28% and 8.42%. The phylum Actinobacteria exhibited a relative abundance of 8.24% in the CJ treatment, positioning it as the fourth most abundant bacterial phylum. In the JXC and WY treatments, its abundance was 6.29% and 6.71%, respectively, making it the fifth most abundant bacterial group. In the CK (control), FJ, and QK treatments, its relative abundances were 6.93%, 6.75%, and 6.51%, respectively, placing it as the sixth most abundant bacterial group. The phylum Planctomycetes showed a relative abundance of 8.09% in the rhizosphere soil of the CJ treatment, making it the fifth most abundant bacterial phylum. The phylum Bacteroidetes had relative abundances of 7.10%, 7.67%, and 7.14% in the rhizosphere soil of the CK (control), FJ, and QK treatments, respectively. In the CJ, JXC, and WY treatments, its relative abundances were 7.73%, 6.28%, and 6.45%, ranking it as the fifth and sixth most abundant bacterial phylum, respectively. Other phyla, including Verrucomicrobia, Chloroflexi, Firmicutes, and Candidatus Dormibacteraeota, had relative abundances below 4.38%, while the remaining phyla accounted for less than 1% of the total microbial community (**Figure 6**).

**Figure 7:**
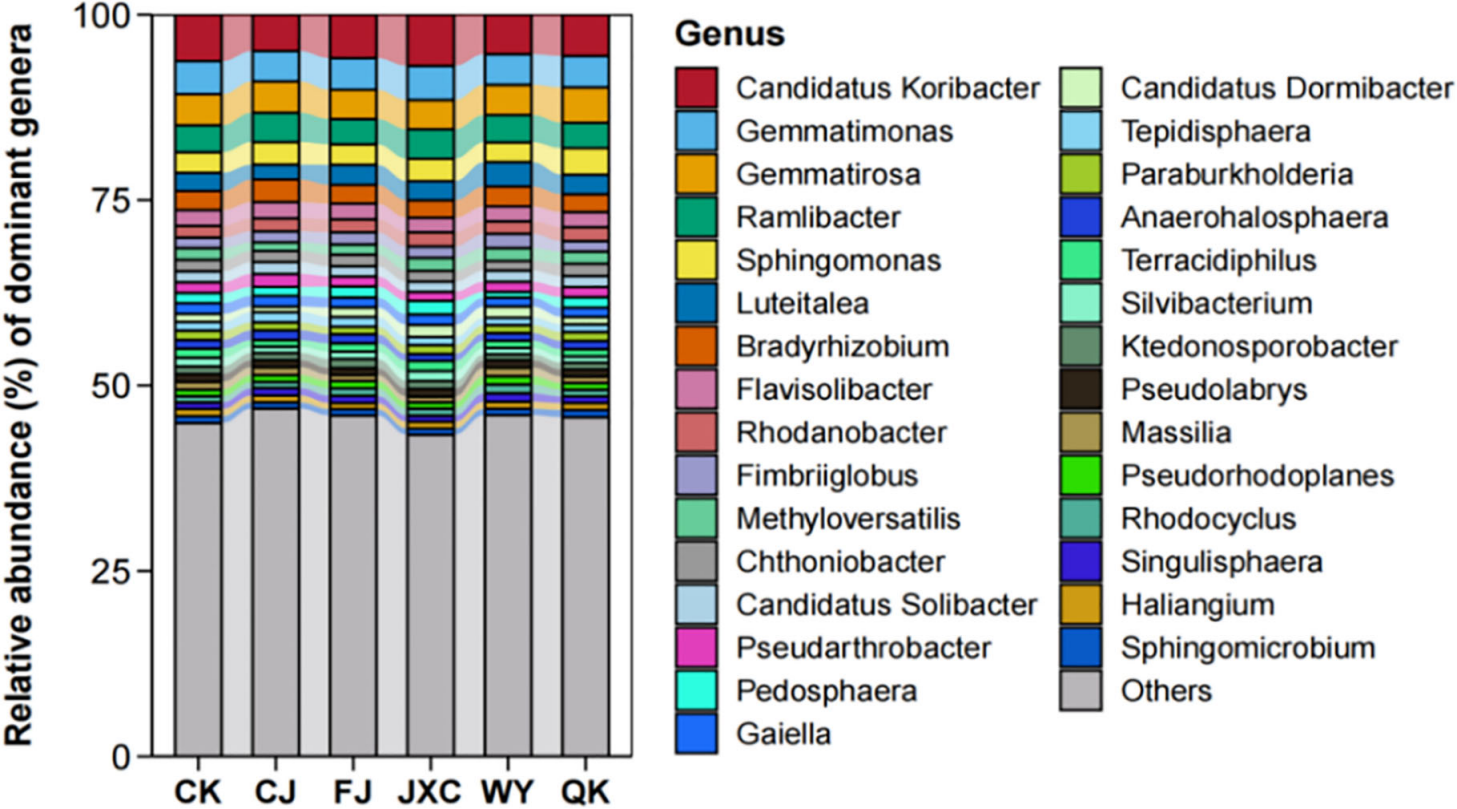
Relative abundance of bacterial communities at the genus level in potato rhizosphere soil during the full flowering stage phylum-level composition and structural analysis of fungal communities in the potato rhizosphere. Treatments include the control (CK), *Streptomyces scabies*, (CJ), *Spongospora subterranea* (FJ), *Globodera rostochiensis* (JXC), *Phytophthora infestans* (WY), and *Ralstonia solanacearum* (QK).

At the genus level, the number of bacterial genera identified in the CK, CJ, FJ, JXC, WY, and QK treatments was 672, 737, 731, 701, 751, and 714, respectively. This indicates that soilborne pathogen treatments slightly increased the diversity of bacterial genera in the potato rhizosphere. The top 15 dominant genera across all treatments included *Candidatus Koribacter, Gemmatimonas, Gemmatirosa, Ramlibacter, Sphingomonas, Luteitalea, Bradyrhizobium, Flavisolibacter, Rhodanobacter, Fimbriiglobus, Methyloversatilis, Chthoniobacter, Candidatus Solibacter, Pseudarthrobacter*, and *Pedosphaera*. Among these, *Candidatus Koribacter* was the most dominant genus, with relative abundances ranging from 4.92% to 6.91% across all treatments. *Gemmatimonas* was the second most abundant genus in the CK, FJ, JXC, and WY treatments, with relative abundances of 4.45%, 4.27%, 4.61%, and 4.13%, respectively, and ranked third in the CJ and QK treatments. Genera such as *Luteitalea, Bradyrhizobium*, and *Flavisolibacter* exhibited relative abundances below 3.05% across all treatments, while seven genera showed relative abundances below 1.93%, and 15 genera were below 1.62% (**Figure 7**).

**Figure 8:**
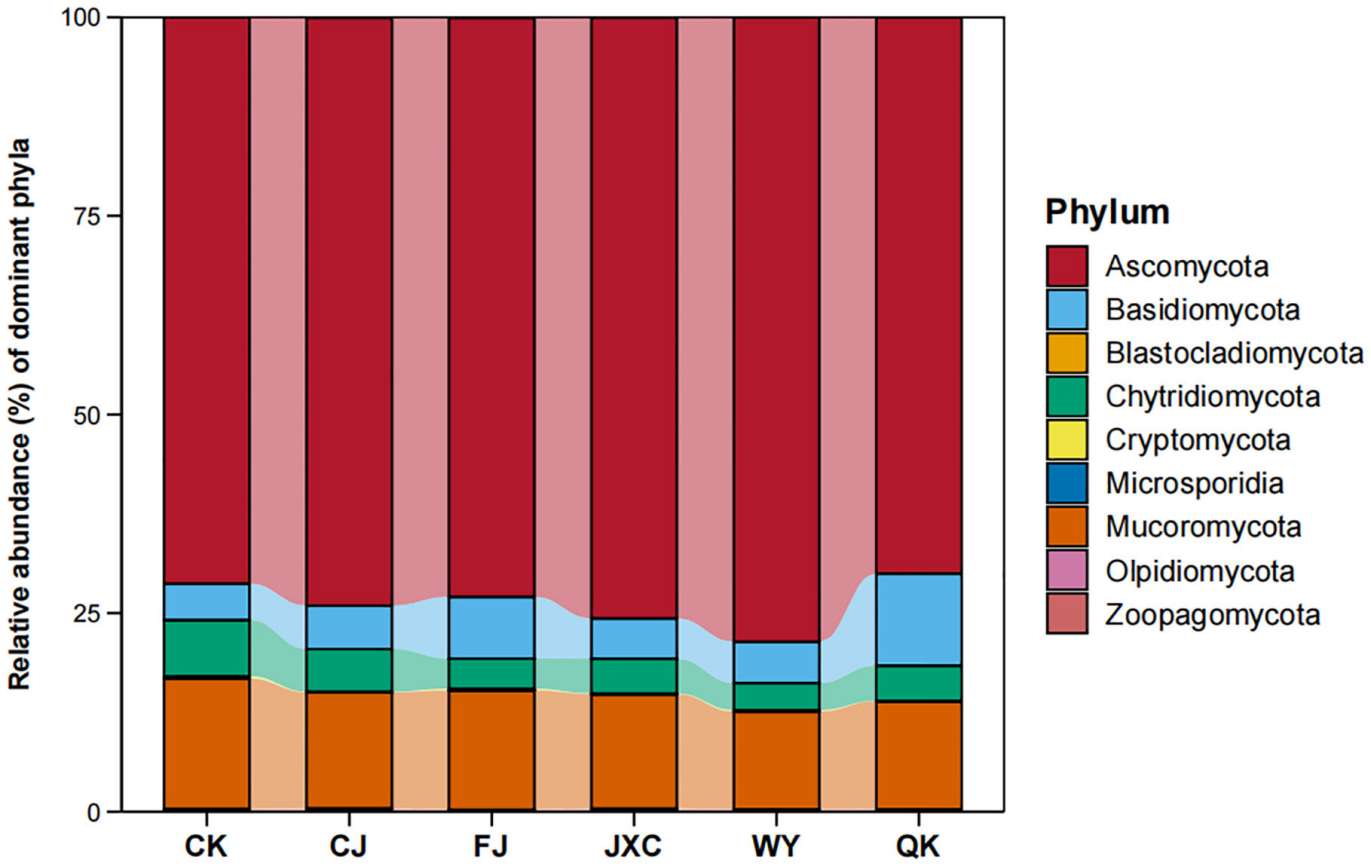
Relative abundance of fungal communities at the phylum level in potato rhizosphere soil during the full flowering stage genus-level composition and structural characteristics of fungal communities in the potato rhizosphere. Treatments include the control (CK), *Streptomyces scabies*, (CJ), *Spongospora subterranea* (FJ), *Globodera rostochiensis* (JXC), *Phytophthora infestans* (WY), and *Ralstonia solanacearum* (QK).

At the phylum level, a total of 10 fungal phyla were identified. The presence of the scab pathogen decreased the number of fungal phyla, while other pathogens had no significant effect on fungal diversity at this level. The dominant fungal communities included *Ascomycota*, *Basidiomycota*, *Blastocladiomycota*, *Chytridiomycota*, *Cryptomycota*, *Microsporidia*, *Mucoromycota*, *Olpidiomycota*, *Zoopagomycota*, and an unclassified phylum, with average relative abundances of 73.70%, 6.61%, 0.01%, 4.74%, 0.14%, 0.02%, 14.37%, 0.01%, 0.33%, and 0.06%, respectively. *Ascomycota* was the most abundant phylum across treatments, with relative abundances ranging from 69.98% to 78.56%. *Mucoromycota* was the second most dominant phylum, with relative abundances between 13.61% and 16.35% across treatments (**Figure 8**).

**Figure 9:**
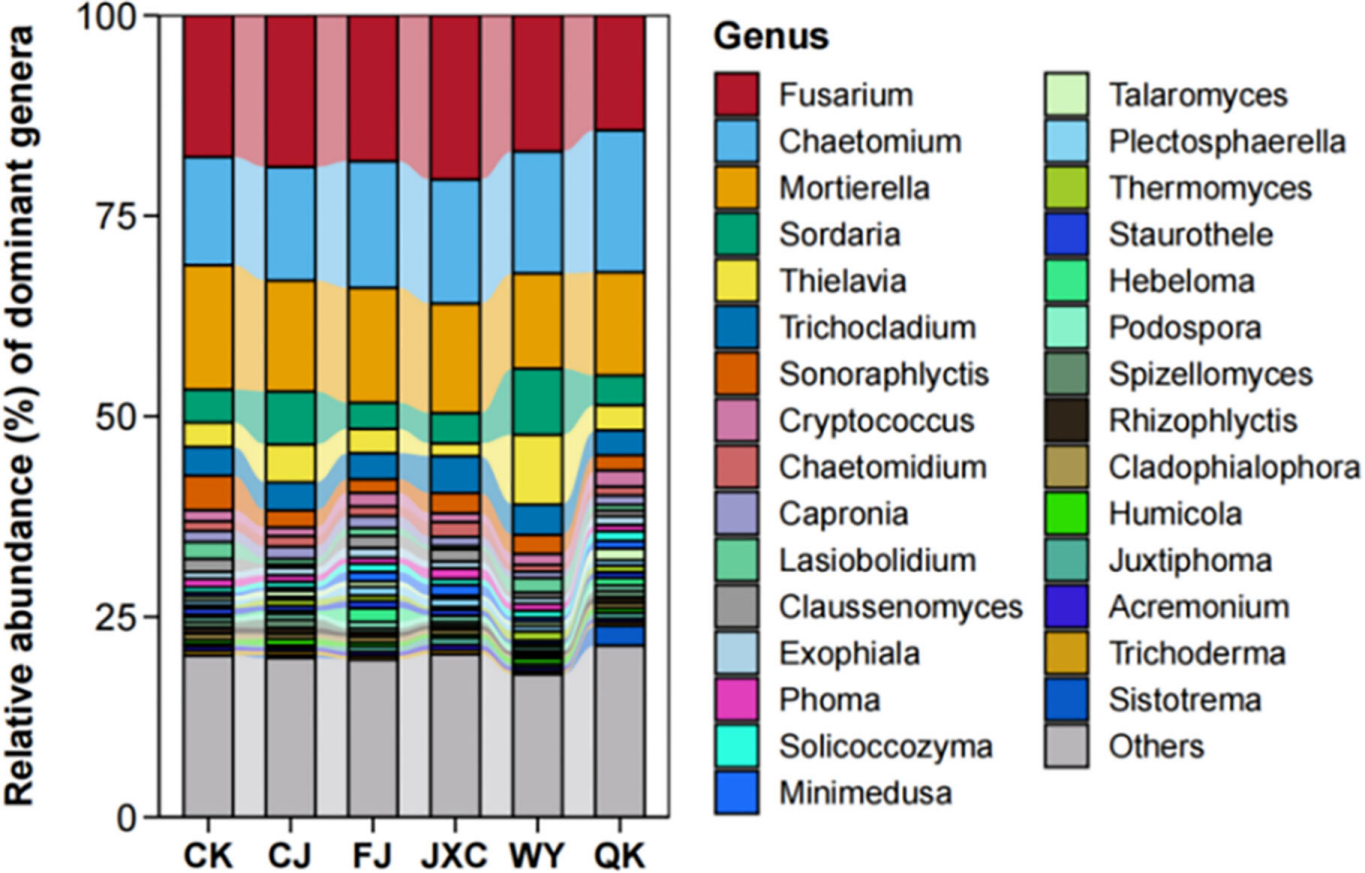
Relative abundance of fungal communities at the genus level in potato rhizosphere soil during the full flowering stage correlation analysis of microbial communities and enzyme activities in the potato rhizosphere soil. Treatments include the control (CK), *Streptomyces scabies*, (CJ), *Spongospora subterranea* (FJ), *Globodera rostochiensis* (JXC), *Phytophthora infestans* (WY), and *Ralstonia solanacearum* (QK).

At the genus level, the number of fungal genera identified in the CK, CJ, FJ, JXC, WY, and QK treatments was 395, 363, 398, 432, 448, and 420, respectively, indicating variations in genus-level species richness among treatments. The presence of the scab pathogen reduced fungal genus richness, while the other four soilborne pathogens increased it. *Fusarium* was the most abundant genus in the control (CK), CJ, FJ, JXC, and WY treatments, with relative abundances of 17.66%, 18.90%, 18.19%, 20.47%, and 16.96%, respectively, while it ranked as the second most abundant genus in the QK treatment, with a relative abundance of 14.33%. *Chaetomium* was the third most abundant genus in the control group, the top genus in the QK treatment, and the second most dominant genus in the other treatments, with relative abundances ranging from 13.49% to 17.70% (**Figure 9**). For Pearson correlation analysis with enzyme activities, the top 10 dominant bacterial and fungal phyla in potato rhizosphere soil during the full bloom stage were selected.

### Correlation between dominant phylum-level microbial communities and enzyme activities

**Figure 10:**
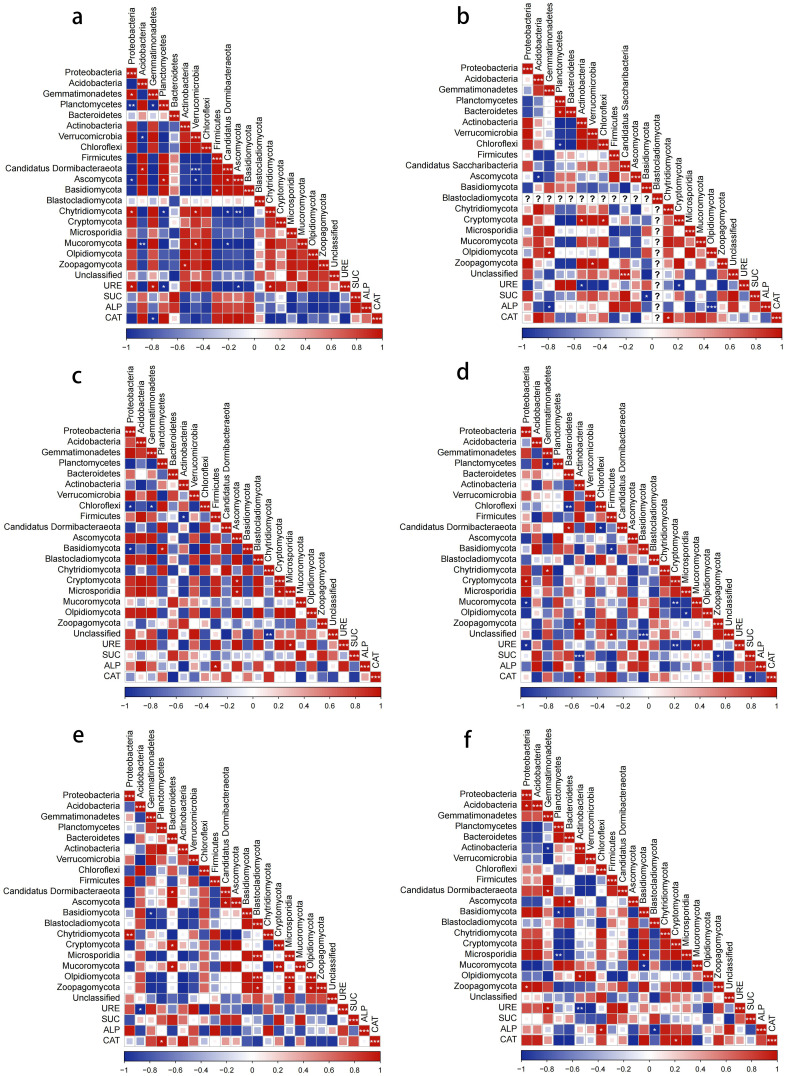
Correlation analysis of bacterial and fungal dominant phylum and enzyme activities with different soilborne pathogens treatment. **(a)** control. **(b)** common scab pathogen. **(c)** powdery scab pathogen. **(d)** nematode. **(e)** late blight pathogen. **(f)** bacterial wilt pathogen.

In the bacterial community, the relative abundance of *Proteobacteria* and *Planctomycetes* in the control group was significantly positively and negatively correlated with URE activity, respectively. The relative abundance of *Gemmatimonadetes* was positively correlated with URE activity and negatively correlated with CAT activity, with all correlation coefficients being 1 (**Figure 10a**). In the scab pathogen treatment, *Actinobacteria* abundance was negatively correlated with URE activity, while *Gemmatimonadetes* was significantly negatively correlated with ALP activity (**Figure 10b**). In the powdery scab pathogen treatment, the relative abundance of *Firmicutes* showed a significant positive correlation with ALP activity (**Figure 10c**). In the nematode treatment, *Proteobacteria* abundance was significantly negatively correlated with URE activity, while *Actinobacteria* was highly negatively correlated with SUC activity (*p* < 0.001) and significantly positively correlated with CAT activity (**Figure 10d**). In the late blight pathogen treatment, *Acidobacteria* abundance was negatively correlated with URE activity, while *Planctomycetes* showed a significant positive correlation with CAT activity (**Figure 10e**). In the bacterial wilt pathogen treatment, URE activity exhibited a significant positive correlation with *Gemmatimonadetes* and a significant negative correlation with *Actinobacteria*. Additionally, ALP activity was significantly positively correlated with *Chloroflexi* abundance (**Figure 10f**).

In the fungal community, there was a significant negative correlation between URE and SUC activities and the relative abundance of Olpidiomycota, as well as between ALP activity and Olpidiomycota in the control group. Under the powdery scab pathogen treatment, the relative abundance of Microsporidia showed a significant positive correlation with URE activity. In the nematode treatment, URE activity was significantly negatively correlated with Cryptomycota and positively correlated with Mucoromycota, while Zoopagomycota was negatively correlated with SUC activity. For the late blight pathogen treatment, no significant correlations were observed between the dominant fungal phyla and soil enzyme activities. In the bacterial wilt pathogen treatment, the relative abundances of Blastocladiomycota and Cryptomycota were significantly negatively and positively correlated with ALP and CAT activities, respectively. Additionally, in the control soil, URE activity in the fungal community was significantly positively correlated with the relative abundance of *Chytridiomycota* and negatively correlated with Ascomycota. Under the scab pathogen treatment, *Chytridiomycota* was positively correlated with CAT activity, while Blastocladiomycota and Cryptomycota were negatively correlated with CAT activity.

### Correlation between dominant microbial communities at the genus level and enzyme activities

**Figure 11:**
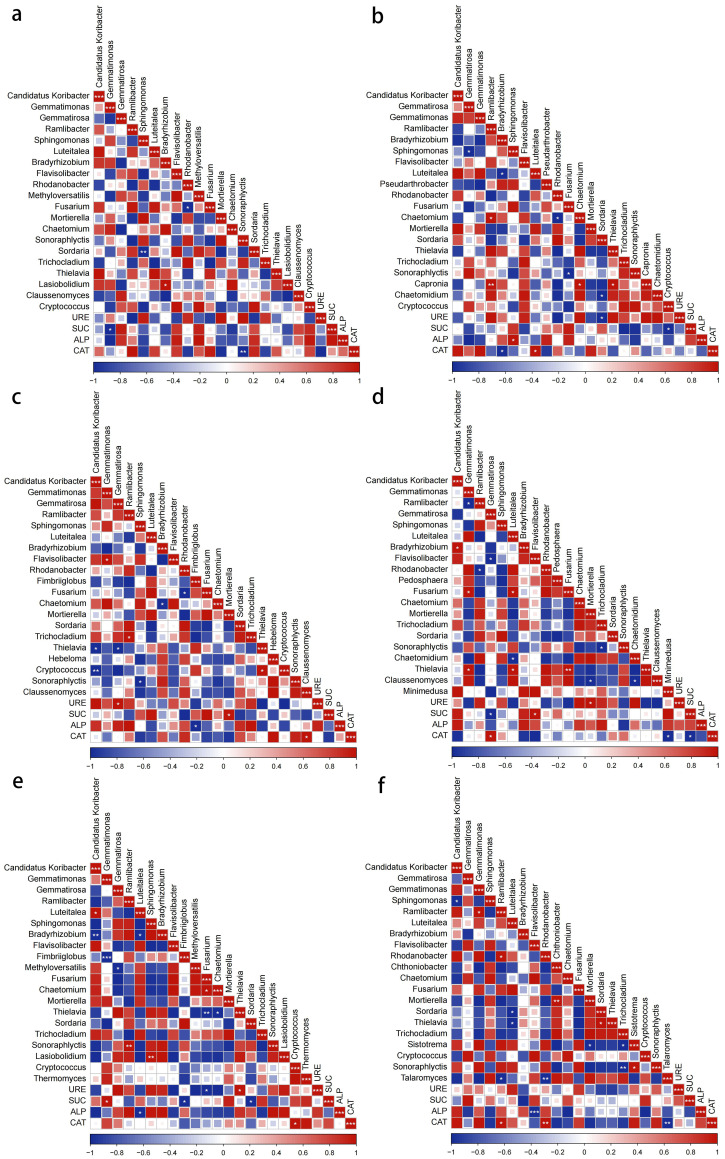
Correlation analysis of bacterial and genus dominant genus and enzyme activities with different soilborne pathogens treatment **(a)** control. **(b)** common scab pathogen. **(c)** powdery scab pathogen. **(d)** nematode. **(e)** late blight pathogen. **(f)** bacterial wilt pathogen.

Pearson correlation analysis revealed significant relationships between microbial genera and enzyme activities, with distinct patterns among treatments. In the bacterial community, the relative abundance of *Gemmatimonas* in the control soil was significantly negatively correlated with SUC activity (p < 0.05, hereafter the same significance level applies; **Figure 11a**). Under the scab pathogen treatment, *Bradyrhizobium* showed a negative correlation with CAT activity, whereas *Sphingomonas* and *Luteitalea* were positively correlated with ALP and CAT activities, respectively (**Figure 11b**). In the powdery scab pathogen treatment, the relative abundance of *Gemmatirosa* was significantly positively correlated with URE activity, while *Fimbriiglobus* abundance showed a significant negative correlation with ALP activity (**Figure 11c**). In the nematode treatment, the relative abundance of *Gemmatirosa* was significantly negatively correlated with SUC activity and positively correlated with CAT activity, while Flavisolibacter was positively correlated with SUC activity (**Figure 11d**). In the late blight pathogen treatment, the relative abundances of *Gemmatimonas* and *Fimbriiglobus* were significantly positively and negatively correlated with SUC activity, respectively, whereas *Luteitalea* abundance was negatively correlated with ALP activity (**Figure 11e**). In the bacterial wilt pathogen treatment, the relative abundances of *Ramlibacter* and *Rhodanobacter* were significantly negatively correlated with CAT activity, while *Flavobacterium* abundance showed a strong negative correlation with ALP activity (**Figure 11f**).

In the fungal genera, *Sonoraphlyctis* in the control soil was significantly negatively correlated with CAT activity. In the scab pathogen treatment, *Sordaria* and *Cryptococcus* were negatively correlated with URE and SUC activities, respectively. In the powdery scab pathogen treatment, *Mortierella* and *Claussenomyces* were positively correlated with SUC and CAT activities, respectively. In the nematode treatment, the relative abundance of *Mortierella* was positively correlated with URE activity, while *Minimedusa* was negatively correlated with CAT activity. In the late blight pathogen treatment, the relative abundances of *Fusarium* and *Thielavia* were significantly negatively and positively correlated with URE activity, respectively, while *Sordaria* abundance was negatively correlated with SUC activity, and *Cryptococcus* was positively correlated with CAT activity. In the bacterial wilt pathogen treatment, *Talaromyces* was significantly negatively correlated with CAT activity (**Figure 7**). In summary, there is a correlation between microbial communities in the potato rhizosphere and enzyme activities. The richness of bacterial communities was primarily associated with URE, ALP, and CAT activities, while fungal communities were mainly correlated with CAT, URE, and SUC activities.

## Discussion

This study provides a comprehensive analysis of the effects of soilborne pathogens on enzymatic activities and microbial dynamics in the potato rhizosphere. The findings reveal that different pathogens influence the rhizosphere environment in unique ways, implicating disease development and management. Reductions in urease and sucrase activities in pathogen-treated soils align with previous studies showing disrupted nutrient cycling. Notably, the increased alkaline phosphatase activity in some pathogen treatments may indicate microbial adaptations to mitigate stress by improving phosphorus mobilization.

Soil enzyme activity serves as a crucial indicator of microbial activity, soil environment, and overall soil quality. Enzymes such as urease, sucrase, alkaline phosphatase, and catalase play vital roles in nutrient cycling and plant growth (Chen et al., 2020). For instance, urease reflects nitrogen availability (Abbas, 2017), while sucrase and alkaline phosphatase are involved in carbon and phosphorus cycling, respectively, facilitating the conversion of organic matter into forms readily absorbed by plants (Wang et al., 2018; Carrara et al., 2018). Catalase, on the other hand, reduces oxidative stress by breaking down hydrogen peroxide. The observed pathogen-induced disruptions in these enzyme activities align with findings from Chelius’s studies on crops like *Panax notoginseng* and tobacco, reinforcing their relevance as indicators of disease dynamics (Chelius and Triplett, 2001).

Rhizosphere is a dynamic and complex environment shaped by intricate interactions between plants and a diverse microbial community (Atif et al., 2024). Plant root exudates serve as essential nutrient sources for rhizospheric bacteria, with small molecular compounds playing a pivotal role in shaping soil microbial community structure. These bioactive molecules not only influence microbial composition but also modulate the functional dynamics of soil microbial communities, thereby impacting soil health and plant-microbe interactions (Gu et al., 2020). The microbial community analysis underscores the complex interplay between pathogens, enzymes, and microbial taxa. Significant shifts in bacterial and fungal diversity were observed under pathogen stress. For example, the enrichment of *Gemmatimonas* and *Flavisolibacter* in powdery scab treatments and *Trichocladium* in late blight treatments suggests their potential roles as disease promoters or indicators. These findings align with studies linking soilborne diseases to specific microbial groups. The presence of pathogens such as *Fusarium* and *Streptomyces* has been associated with either promoting or mitigating disease, depending on the context. Previous studies have shown that soilborne pathogens affect enzyme activities in a pathogen- and crop-specific manner. For example, soils affected by root rot in *Panax notoginseng* compared to healthy soils (You et al., 2014), whereas urease activity is markedly lower in soils with tobacco wilt in healthy soils (Wang et al., 2017). Similar trends were observed in soils with *Astragalus* root rot, where reduced urease activity and increased cellulase and sucrase activities were linked to enhanced microbial biomass and carbon metabolism (Zhao, 2022). The specific effects of soilborne pathogens on potato rhizosphere enzyme activities highlight the need for more targeted research to understand crop-specific interactions.

Soilborne pathogens altered the activities of urease, sucrase, alkaline phosphatase, and catalase over time. However, the results differed from previous findings, potentially due to differences in crop and pathogen types. Few studies have specifically examined the effects of potato diseases on rhizosphere soil enzyme activities. This study measured only four soil enzymes; future studies should investigate additional enzymes and other indicators to provide a more comprehensive understanding of disease development.

Research indicates that soilborne diseases in plants are closely associated with specific microbial groups, which can act as indicators of soil health or predictors of disease occurrence. Zhang et al. (2023) reported that the presence of clubroot in cabbage significantly increased the abundance of *Fusarium, Gibberella, Kernia*, and *Thielavia* in the rhizosphere. Similarly, Han et al. (2017) found a significant negative correlation between the incidence of millet smut and the abundance of *Streptomyces* and *Bradyrhizobium* in the soil. Additionally, root rot in Panax notoginseng has been shown to reduce the abundance of beneficial soil bacteria, such as *Bacillus* and *Streptomyces* spp., which play a critical role in disease prevention and control (Lu et al., 2015).

Pearson correlation analysis revealed significant relationship between microbial communities and disease incidence. While no significant correlations were observed in soils infested with common scab pathogen, bacterial wilt pathogen, or nematodes, a positive correlation was found between powdery scab incidence and the relative abundances of *Gemmatimonas* and *Flavisolibacter.* These bacterial taxa may support pathogen proliferation by converting nitrogen into amino compounds or secreting antibiotics or hormone-like substances that suppress or eliminate harmful microorganisms (Zhou et al., 2022). Similarly, the abundance of *Trichocladium* was positively correlated with late blight incidence, suggesting its involvement in pathogen dynamics. These microbial groups could serve as potential indicators of soil health and predictors of powdery scab or late blight in potatoes. Although further research is needed to fully elucidate their specific roles in the soil ecosystem and their interactions with pathogens.

While insightful, this study has limitations. The focus on four key enzymes provides only a partial understanding of the enzymatic dynamics in pathogen-stressed soils. Future research should investigate additional enzymes, such as cellulase and dehydrogenase, to gain a more comprehensive view of soil biochemical processes. Furthermore, advanced techniques like functional metagenomics and metabolomics could provide deeper insights into the mechanisms underlying pathogen-induced changes in rhizosphere processes. These approaches would enhance our understanding of how pathogens influence soil biochemical and microbial dynamics, ultimately contributing to more effective disease management strategies.

## Conclusion

In conclusion, this study demonstrates that soilborne pathogens profoundly influence rhizosphere enzymatic activities and microbial dynamics, thereby affecting nutrient cycling and disease development in potatoes. The observed alterations in urease, sucrase, alkaline phosphatase, and catalase activities suggest a pathogen-specific disruption of soil biochemical processes. The correlations between microbial taxa and disease incidence highlight the potential of using microbial profiles as indicators for soil health and disease prediction. Our results demonstrate that certain microbial consortia improve soil structure, increase nutrient availability, and stimulate plant immune responses, thereby creating an unfavorable environment for pathogenic organisms. Regarding the pathogens targeted, our findings indicate significant suppression of *Phytophthora*, which are major contributors to soilborne diseases. These effects were observed through the prevalence of potato scab and late blight diseases exhibits significant correlations with specific microbial taxa. The incidence of potato scab is negatively correlated with the abundance of *Planctomycetes* and *Basidiomycota* but positively correlated with the abundance of *Gemmatimonadetes* and *Lysobacter*. In contrast, the occurrence of late blight disease shows a significant positive correlation with the abundance of *Ascomycota*, and *Brevibacillus*. These findings provide valuable insights for developing sustainable disease management strategies, such as leveraging beneficial microbial communities to restore soil health and suppress pathogens. Ultimately, this research contributes to the understanding of pathogen-rhizosphere interactions and lays the groundwork for integrated approaches to enhance potato productivity and soil sustainability.

## Data Availability

The original contributions presented in the study are publicly available. This data can be found here:https://www.ncbi.nlm.nih.gov/sra/?term=PRJNA1251261/PRJNA1251261.
